# Predictors of failure of DISE-directed adenotonsillectomy in children with sleep disordered breathing

**DOI:** 10.1186/s40463-017-0213-3

**Published:** 2017-05-05

**Authors:** Noura Alsufyani, Andre Isaac, Manisha Witmans, Paul Major, Hamdy El-Hakim

**Affiliations:** 1 Division of Oral Medicine and Pathology, School of Dentistry, University of Alberta, 5-531 Edmonton Clinic Health Academy, 11405 87 Ave NW, Edmonton, AB T6G 1C9 Canada; 2 Division of Otolaryngology-Head and Neck Surgery, Department of Surgery, University of Alberta, 2C3.57 Walter MacKenzie Centre, 8440 112 St NW, Edmonton, AB T6G 2R7 Canada; 3 Department of Pediatrics, Stollery Children’s Hospital, 76A-80 Chippewa Rd, Sherwood Park, Edmonton, AB T8A 4W6 Canada; 4 Division of Orthodontics, School of Dentistry, University of Alberta, 5-478, Edmonton Clinic Health Academy, 11405 87 Ave, Edmonton, AB T6G 1C9 Canada; 5 Division of Pediatric Surgery, Department of Pediatrics, The Stollery Children’s Hospital, 2C3.57 Walter MacKenzie Centre, 8440 112 St NW, Edmonton, AB T6G 2R7 Canada; 6 Department of Oral Medicine and Diagnostic Sciences, College of Dentistry, King Saud University, Riyadh, Saudi Arabia

**Keywords:** Sleep disordered breathing, Obstructive sleep apnea, Adenotonsillectomy, DISE, Children

## Abstract

**Background:**

Adenotonsillectomy (AT) is the most commonly performed procedure for sleep disordered breathing (SDB) in pediatrics. However, 20-40% of patients will have persistent signs and symptoms of SDB after AT. Drug-induced sleep endoscopy (DISE) has the potential to individualize surgical treatments and avoid unnecessary or unsuccessful surgeries. The objective of this study was to determine the predictors of failure of DISE-directed adenoidectomy and/or tonsillectomy in otherwise healthy children with SDB.

**Methods:**

We retrospectively reviewed a prospective database of children who presented with SDB. All patients underwent preoperative pulse oximetry (PO), followed by DISE with T ± A, The variables documented included demographics, ethnicity, co-morbidities, family history, McGill Oximetry Score (MOS) on PO, as well as findings of collapse and or obstruction on DISE and symptom resolution based on modified Pediatric Sleep Questionnaire (PSQ). The primary outcome was the independent predictors of treatment failure based on multivariate binary logistic regression.

**Results:**

Three hundred eighty-two patients satisfied the inclusion criteria. Based on post-operative modified PSQ, SDB resolved in 259 patients (68%), whereas 123 (32%) had persistent symptoms. On bivariate analysis, neuropsychiatric diagnosis (*r* = 0.286, *p* = 0.042), history of sleepwalking or enuresis (*r* = 0.103, *p* = 0.044), MOS (*r* = 0.123, *p* = 0.033), presence of DNS (*r* = 0.107, *p* = 0.036), and presence of laryngomalacia (*r* = 0.122, *p* = 0.017) all positively correlated with treatment failure. Small tonsil size on DISE correlated with treatment failure (*r* = −0.180, *p* < 0.001). Multivariate analysis identified age greater than 7 years (OR = 1.799, [95% CI 1.040–3.139], *p* = 0.039), obesity (OR = 2.032, [95% CI 1.043–3.997], *p* = 0.040), chronic rhinitis (OR = 1.334, [95% CI 1.047–1.716], *p* = 0.025), deviated nasal septum (OR = 1.745, [95% CI 1.062–2.898], *p* = 0.031) and tonsil size (OR = 0.575, [95% CI 0.429–0.772], *p* < 0.01) as independent predictors of treatment failure.

**Conclusions:**

Obese, asthmatic, and children older than seven years are at increased risk of treatment failure after DISE-directed AT. Several DISE findings can independently predict AT failure, including tonsil size, degree of chronic rhinitis, and the presence of a deviated nasal septum, and can be addressed at a second stage. Further research is needed into the role of DISE in surgically naïve patients with SDB, and to compare DISE-directed surgery with the current standard of care.

## Background

Sleep-disordered breathing (SDB), a clinical spectrum ranging from simple snoring to obstructive sleep apnea (OSA), is a highly prevalent condition that affects 4–11% of children [1, 2]. Its detrimental health effects including learning difficulties, behavioral issues, growth retardation, hypertension, cor pulmonale, and compromised quality of life are well documented [3]. Adenotonsillar hypertrophy has been widely accepted as its most common cause of in children; as a result, adenotonsillectomy (AT) has become one of the most frequently performed operations in North America [3, 4]. In fact, AT is decidedly advocated as the first line treatment for childhood SDB by the American Academy of Pediatrics [5].

However it is estimated that 20–40% of patients will have persistent symptoms and/or signs of SDB after AT [2, 3, 6, 7]. Given the substantial volume of surgeries and the absence of evidenced based criteria to direct surgical treatment, a valid question remains: should all children with SDB have adenoidectomy *and/or* tonsillectomy as the first-line therapy, or is it possible to predict surgical failures and tailor the management? To date, AT non-responders have largely not been accounted for. Limited studies have suggested that variables such as obesity, asthma, severe apnea hypopnea index (AHI) and male gender conferred risk for persistent disease after AT [2, 6].

Drug-induced sleep endoscopy (DISE) is a relatively novel tool that has been advocated to identify surgical targets in children with SDB and predict AT non-responders. It involves an endoscopic assessment of the upper (and sometimes lower) airway during pharmacologically induced sleep. In adults it has been claimed to identify dynamic sites of pathology and severity of airway obstruction [8],. Most pediatric studies have focused on special populations and/or children who had had airway surgery, rather than surgically naïve patients [9, 10].

To date, no studies have examined both patient and DISE related findings as potential predictors of failure of SDB surgery in pediatrics. The purpose of this study was to explore the clinical and DISE variables that may predict failure of DISE-directed adenoidectomy and/or tonsillectomy in otherwise healthy children with SDB.

## Methods

We performed a retrospective review of a prospectively collected surgical database at a tertiary care pediatric center (Stollery Children’s Hospital) between January 2005 and January 2012. The eligible subjects were children (≤17 years old) consecutively diagnosed with SDB. This retrospective review was taken from a cohort of patients who all had adenoidectomy and/or tonsillectomy. It is the surgeon’s practice to perform DISE before any procedure for SDB, and to use the information gained from DISE to direct the next steps of the operation (or lack thereof). Patients (and/or parents) are consented for DISE with possible adenotonsillectomy. If the adenoids and/or tonsils are found to be contributing to the obstruction on DISE, then they are removed as part of the same operation. If another surgical or medical target is identified, it is documented and used for parental counseling, and is also used for planning a second operation should the patient fail to resolve. It should be noted that patients that had no operation based on DISE findings or had a different operation than adenotonsillectomy were not included here.

Included patients must have had a clinical diagnosis of SDB including an abnormal (>33%) modified Pediatric Sleep Questionnaire (PSQ) [11] and physical examination for inclusion. All patients underwent pre-operative pulse oximetry (PO). Since patients had post-operative PO and/or polysomnography (PSG) only if clinically indicated, these endpoints were only used as a secondary outcomes measure. Patients were excluded if they had previous surgical management for SDB, any craniofacial syndromes or dysmorphic features, or any associated genetic, neurologic, muscular or metabolic conditions that would affect sleep patterns.

Pre-operative variables collected included responses to a modified 22-item PSQ [11], as well as a list of inquiries including snoring and its duration (>12 months, 5–7 nights/week), obesity (>97^th^ percentile), pre-maturity at birth (<36 weeks gestation), neuro-psychiatric diagnosis (general developmental delay, autism, attention deficit and hyperactivity disorder, etc.), prior intubation for ventilation in intensive care unit, allergy, asthma, swallowing dysfunction (according to clinical or instrumental testing), parental smoking, and family history of SDB. PO was performed according to previously published standards [12], and the graded according to the McGill Oximetry Score (MOS) [12].

DISE was performed under sedation using total intravenous anesthesia of propofol and fentanyl allowing spontaneous respiration [13]. A flexible neonatal bronchoscope was used for all DISE procedures. Although several previous studies have used internal grading scales to report and score findings on DISE, there exists no widely accepted standard reporting method. The reporting method used in this study is shown in Table 1. The emphasis of this system is to guide the decision to operate rather than merely assign a grade and it had been validated in a previous study (K = 0.83 [0.69–0.98]) [13]. The endoscopic rhinitis score (ERS) is a system to grade the degree of chronic rhinitis using nasal endoscopy which has shown good intra-rater and inter-rater reliability in a previous study [14]. Other reported variables include the degree of deviated nasal septum (DNS), obstructive adenoids [14], presence or absence of nasal polyps, tonsillar obstruction, pharyngeal collapse (lateral wall, antero-posterior, or circumferential), lingual tonsil hypertrophy, and laryngeal findings (laryngomalacia (LM), cleft, stenosis, paralysis). It should be stressed that the tonsil size reported in this study was that identified at the time of DISE, (degree the tonsils obstruct the oropharyngeal airway during simulated sleep), rather than the office based scales. We found only a moderate agreement between the tonsil size documented using the Brodsky scale and that using DISE (K = 0.44; [0.33–0.55]) on a sample of 248 children (unpublished data).

**Table 1:** Scoring system for drug-induced sleep endoscopy (DISE)

Anatomic site/feature	Score
ERS [14]	0–3
DNS	None, <50%, >50%
Nasal polyps	No, Yes
Adenoids	Retropalatal vs.choanal <25%, 25–50%, 50–75%, >75%
Tonsils	<50%, >50%
Pharynx	No collapse, lateral wall, circumferential, anteroposterior, tongue base
Lingual tonsil hypertrophy	No, Yes
Larynx	Normal, malacia, edema, paralysis, late recovery abduction, cleft

*ERS* Endoscopic rhinitis score, *DNS* Deviated nasal septum

### Outcome measures and statistical analyses

The primary outcome was the independent predictors of treatment failure based on parental report of resolution of symptoms of SDB based on modified PSQ (resolved = PSQ <33%; persistent = PSQ > 33%). Secondary outcomes included the change in MOS grades where available.

Basic descriptive statistics of the patient cohort were performed. Data analysis was performed using bivariate correlations (chi-squared tests for categorical data and Pearson’s correlation coefficients for numerical data) between each predictor variable and the primary outcome. Two multivariate binary logistic regressions were then performed, the first with the dependent variable being symptom resolution on PSQ (<33%), the second with the dependent variable being post-operative MOS for patients that had post-operative PO, in order to determine independent predictors of AT failure. For the second analysis, treatment failure was defined as abnormal post-operative MOS (MOS >1). All clinically significant predictor variables as well as variables with univariate correlation *p*-value <0.20 were included in the multivariate regression. Age as a predictor variable was dichotomized to less or more than seven years old to be consistent with other similarly designed studies [2, 6].

## Results

**Fig. 1:**
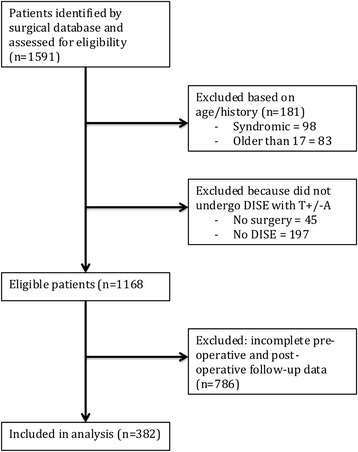
Flow diagram of included/excluded patients

We identified 1591 cases of SDB between January 2005 and 2012. Of these, 98 were syndromic, 242 did not undergo DISE with tonsillectomy and/or adenoidectomy, and 83 were older than 17 (see Fig. 1). Of the remaining 1168 cases, 382 had complete pre-operative clinical data, and post-operative follow-up information available and were included in the study. The median post-operative follow-up period was four months (range 3.0–7.2 months).

The demographic information of the patient cohort is shown in Table 2. The mean age was 6 ± 2.7 years (range 0.5–14.2 years), with 59% males. The obesity rate was 13% and the prevalence of asthma was 11%. On pre-operative PO, the majority were MOS 1 (73%). 207 patients underwent AT, 74 underwent tonsillectomy only, and 101 underwent adenoidectomy only; all DISE guided surgery. Based on post-operative modified PSQ, 259 patients (68%) had resolution of SDB, whereas 123 (32%) had persistent disease. Eighty-six patients had post-operative PO, all of which had abnormal pre-operative PO (MOS >1). Upon subgroup analysis, 41% of this group had an abnormal PSQ (>33%) but only 15% had abnormal PO (failure to transform to MOS 1), likely due to the inherent limited sensitivity of the test (PO).

**Table 2:** Pre-operative characteristics and variables of the patient cohort

Variable	Total (*n* = 382)	AT (*n* = 207)	T (*n* = 74)	A (*n* = 101)
Mean Age in yrs ± SD (range)	6.0 ± 2.7 (0.7–14.2)	5.9 ± 2.5 (1.7–12.9)	6.6 ± 3.0 (0.7–14.2)	5.9 ± 2.8 (1.1–12.7)
Males (%)	225 (59%)	124 (60%)	35 (47%)	66 (65%)
Obesity	51 (13%)	26 (13%)	11 (15%)	14 (14%)
Asthma	43 (11%)	21 (10%)	12 (16%)	10 (10%)
MOS				
1	279 (73%)	145 (70%)	64 (86%)	68 (67%)
2–4	103 (27%)	62 (30%)	10 (14%)	33 (33%)

*AT* Adenotonsillectomy, *T* tonsillectomy, *A* adenoidectomy, *MOS* McGill Oximetry Score

The results of the univariate analysis are shown in table 3. The presence of neuropsychiatric diagnosis (*r* = 0.286, *p* = 0.042), history of sleepwalking or enuresis (*r* = 0.103, *p* = 0.044), MOS (*r* = 0.123, *p* = 0.033), presence of a deviated nasal septum (DNS) (*r* = 0.107, *p* = 0.036), and presence of laryngomalacia (*r* = 0.122, *p* = 0.017) all positively correlated with treatment failure. Tonsil size negatively correlated with treatment failure (*r* = −0.180, *p* < 0.001), meaning that patients with larger tonsils on endoscopy were more likely to have symptom resolution upon surgery.

**Table 3:** Univariate analysis of correlation between clinical/DISE variables and symptom resolution

Variable	n (%)	Correlation coefficient	*P*-value
Age >7	95 (25%)	0.061	0.238
Male Gender	225 (59%)	0.052	0.312
Ethnicity	N/A	0.020	0.697
Asthma	43 (11%)	0.020	0.690
Allergy	32 (8%)	0.014	0.784
Obesity	51 (13%)	0.059	0.250
Neuropsychiatric Diagnosis	22 (6%)	0.286	0.042*
Sleepwalking/Enuresis	113 (30%)	0.103	0.044*
MOS	N/A	0.123	0.033*
ERS	N/A	0.096	0.061
DNS	N/A	0.107	0.036*
Tonsil Size	N/A	−0.180	<0.001*
Adenoid Size	N/A	−0.023	0.653
Pharyngeal Collapse	115 (30%)	0.024	0.639
Lingual Tonsil Hypertrophy	29 (8%)	−0.007	0.889
LM	24 (6%)	0.122	0.017*

*MOS* McGill Oximetry Score, *ERS* Endoscopic rhinitis score, *DNS* deviated nasal septum, *LM* laryngomalacia

The results of the multivariate logistic regression analysis using treatment failure as the dependent variable and including all patients are shown in Table 4. There were several variables that predicted independently treatment failure. These included age greater than 7 (OR = 1.799, [95% CI 1.040–3.139], *p* = 0.039), obesity (OR = 2.032, [95% CI 1.043–3.997], *p* = 0.040), ERS (OR = 1.334, [95% CI 1.047–1.716], *p* = 0.025), DNS (OR = 1.745, [95% CI 1.062–2.898], *p* = 0.031) and tonsil size (OR = 0.575, [95% CI 0.429–0.772], *p* < 0.01). No other clinical or DISE variables were significant independent predictors of post-operative treatment failure, although there was a non-statistically significant relationship between LM and treatment failure (OR = 2.440 [95% CI 0.995–5.979], *p* = 0.051). Of the patients who failed treatment, 89% (*n* = 109) had an alternative diagnosis on DISE aside from adenotonsillar hypertrophy. The most common alternate diagnoses were inferior turbinate hypertrophy (*n* = 77), pharyngeal collapse (*n* = 39), DNS (*n* = 15), LM (*n* = 13), and lingual tonsil hypertrophy (*n* = 9).

**Table 4:** Multivariate logistic regression of clinical/DISE variables and treatment failure based on symptom resolution

Variable	Exp. (B) [95% CI]	*P*-value
Age >7	1.799 [1.040–3.139]	0.039
Obesity	2.032 [1.043–3.997]	0.040
Tonsil Size	0.575 [0.429–0.772]	<0.001
ERS	1.334 [1.047–1.716]	0.025
DNS	1.745 [1.062–2.898]	0.031
LM	2.440 [0.995–5.979]	0.051

*ERS* Endoscopic rhinitis score, *DNS* deviated nasal septum, *LM* laryngomalacia

The second multivariate regression was performed on patients that had post-operative PO performed based on clinical reasons (*n* = 86, Table 5). The only independent predictors of treatment failure based on post-operative MOS were obesity (OR = 6.699 [95% CI 1.710–26.232], *p* = 0.003) and asthma (OR = 7.572 [95% CI 1.296–44.421], *p* = 0.025).

**Table 5:** Binary multivariate logistic regression of clinical/DISE variables and treatment failure based on post-operative MOS (*n* = 86)

Variable	Exp. (B) [95% CI]	*P*-value
Age >7	0.147 [0.059–1.527]	0.147
Obesity	6.699 [1.710–26.232]	0.003
Asthma	7.572 [1.296–44.241]	0.025

*MOS* McGill Oximetry Score

## Discussion

SDB is a heterogeneous disease in children and although AT is the officially endorsed and most frequently used surgical management, it is not curative in many instances. Much like asthma, we believe that there will be some phenotypic considerations that will emerge as we continue to learn more about the dynamic airway in sleep disordered breathing. Our results demonstrated a 32% failure rate in resolving symptoms based on modified PSQ, which is similar to the rates reported in other studies [2, 15]. The uniqueness of this study was combined use of DISE related variables and parent/patient reported outcome, particularly as the latter does not necessarily follow normalization of sleep studies parameters. It is important to note that for the group that had post-operative PO, treatment failure based on MOS and treatment failure based on symptoms were very different (15 and 41% respectively), which demonstrates that objective improvements in sleep data may not reflect symptom resolution.

The demonstration that older and obese children were more likely to fail treatment is in keeping with previous studies, as is the demonstration that asthma is an independent predictor of treatment failure based on post-operative MOS [2, 6]. Tauman et al. reported that obese children were twice as likely to have abnormal post-operative AHI than non-obese counterparts [6]. However, identifying DNS and severe ERS as additional independent predictors of failure is novel [16]. This suggests, and intuitively, that intra-nasal pathology other than adenoid hypertrophy can be implicated in childhood SDB, and should be sought in a significant number of children. These findings have also been supported in a large retrospective study that found similar results [2].

The results also included an inverse predictive relationship between the tonsil size and treatment failure. This is not surprising, as children with large obstructive tonsils should have symptom improvement after surgery. As previously alluded to, the tonsil size here was that documented upon DISE, rather than an in-office grading. As we mentioned earlier, the agreement between the two is far from perfect. This is may help interpret the results of Tang et al. who studied 70 children who had undergone AT claiming that tonsil size did not correlate with apnea hypopnea index, and that symptomatic improvement had been documented in non obstructive tonsils as per the Brodsky scale [17]. This should be an impetus to compare in future work the size of tonsils using both methods, and counsel against committing the child for potentially unnecessary surgery based solely on traditional trans-oral exam. The nature of the examination in the office does not allow the clinical to determine or predict the nature of the airway collapse during sleep, therefore DISE provides an opportunity to assess the airway in more detail.

Ours is the first study of surgically naive children and to explore DISE variables that may be predictive of AT treatment failure. Aside from the findings of DNS, ERS and tonsil size discussed above, there was a non-statistically significant relationship with presence of LM and treatment failure (but only 8% had LM). Since we only included patients that had AT or one of them, we cannot accept this failure rate as a global one for DISE directed surgeries. Rather we actually claim that DISE will demonstrate an alternate surgical solution should the traditional procedure (here adenoidectomy and/or tonsillectomy) fail partially or is deemed unnecessary in part, and alert to the potential need for other non-surgical options and prepare the parents and the child for that. In this study 89% of those who failed had an alternate diagnosis on DISE (significant DNS, LM, lingual tonsil hypertrophy, or inferior turbinate hypertrophy).

It should be stressed that the patient cohort analyzed in this study automatically excluded patients who were evaluated for SDB, underwent DISE and based on the DISE findings did *not* undergo adenotonsillectomy. A significant proportion of this group are those younger than age three and are an interesting group to study in their own right as their DISE findings tend to be unique and AT is often unsuccessful [18–21]. The exclusion of such groups here and in other studies may have missed to demonstrate a bimodal peak for age as a predictor. We plan to study this group specifically in a future study.

Previous studies that examined the use of DISE in children with SDB only included patients with residual symptoms and were previously treated with surgery [22, 23]. These studies identified occult LM and lingual tonsil hypertrophy as potential causes of AT failure [24]. Other studies also examined special populations (ex: syndromic, neurologically impaired, etc.) [9, 10]. DISE in surgically naive patients had only been reported in adults, and has shown utility in changing management decisions [25–27], as well as improving surgical outcomes for SDB [28, 29]. Overall, our results suggest such utility may indeed exist in pediatric SDB. One may surmise that had the sample size been larger, and all DISE directed surgeries been included the impact of laryngeal and tongue base problems might have been more significant.

This study has some limitations including the retrospective design, and a considerable attrition of data (382 patients had complete information out of a total 1168). We also combined all patients that had adenoidectomy, tonsillectomy, or AT. But since the patients excluded due to absent data are similar (non-complex, non-syndromic children) the main impact will be on sample size limitations that may have down played the significance of some subgroups (ex: LM, lingual tonsil hypertrophy, neuropsychiatric disorders). These results nevertheless highlight some of the challenges faced by clinicians in deciding optimal treatments for children with SDB.

In the future, we hope to gather more data prospectively, as well as perform a decision analysis to determine whether DISE changes management decisions in pediatric SDB. We also hope to compare outcomes of DISE-directed surgery against the current standard of care.

## Conclusions

Despite its widespread use for SDB, more than 30% of patients will fail to resolve symptomatically after AT. Obese patients, asthmatics, and children older than seven years are at increased risk of treatment failure after DISE-directed AT. Several DISE findings can independently predict AT treatment failure, including tonsil size, degree of ERS, and the presence of DNS. DISE can also be used to inform the need for and target of a second stage procedure. Further research is needed into the role of DISE in surgically naïve patients with SDB, and to compare DISE-directed surgery with the current standard of care.

## Abbreviations

AHI: Apnea hypopnea index
AT: Adenotonsillectomy
DISE: Drug-induced sleep endoscopy
DNS: Deviated nasal septum
ERS: Endoscopic rhinitis score
LM: Laryngomalacia
MOS: McGill oximetry score
OSA: Obstructive sleep apnea
PO: Pulse oximetry
PSG: Polysomnography
PSQ: Pediatric sleep questionnaire
SDB: Sleep-disordered breathing
